# Development of a Conceptual Model to Understand Disease Burden in Patients With Systemic Lupus Erythematosus and Organ Damage

**DOI:** 10.36469/001c.82228

**Published:** 2023-08-18

**Authors:** Lynne Broderick, Wen-Hung Chen, Roger A. Levy, April Mitchell Foster, Cindy Umanzor Figueroa, Kerry Gairy, Deven Chauhan

**Affiliations:** 1 QualityMetric, Johnston, Rhode Island, USA; 2 GSK, Value Evidence and Outcomes, Collegeville, Pennsylvania, USA; 3 GSK, Global Medical Affairs, Collegeville, Pennsylvania, USA; 4 GSK, Value Evidence and Outcomes, Brentford, Middlesex, UK

**Keywords:** concept elicitation, systemic lupus erythematosus, qualitative research, organ damage, conceptual model, burden of disease

## Abstract

**Background:** Systemic lupus erythematosus (SLE) is a chronic autoimmune disease that can lead to irreversible organ damage (OD). Data describing the patient burden of OD, as compared with SLE without OD, are limited. **Objective:** To develop a comprehensive conceptual model describing the burden experienced by patients living with SLE-associated OD. **Methods:** There were three phases to this qualitative study. First, a targeted literature review was conducted to inform a draft conceptual model. Second, key opinion leaders (KOLs) were interviewed to assess the draft conceptual model and help shape patient interview materials. Third, patients of different demographic backgrounds from across the United States were interviewed individually to gather their perspectives on living with SLE-associated OD. Data from concept elicitation interviews with KOLs and patients were coded and analyzed using NVivo software to identify the key concepts of the overall patient burden of SLE-associated OD. Findings from the KOL and patient interviews were used to finalize the conceptual model. **Results:** KOLs highlighted that SLE-associated OD carried a higher rate of mortality than SLE alone. Participants with SLE-associated OD (n = 40) experienced detrimental impacts across 4 areas of their lives: physical, cognitive, psychosocial functioning, and economic and work-related well-being. Physical impacts were described by all participants, often affecting their ability to perform everyday tasks. Many also described deterioration of cognitive functioning. Almost all participants experienced emotional impacts and challenges to their relationships and social lives resulting from living with SLE-associated OD. Additionally, SLE-associated OD imposed an economic burden including increased healthcare costs. SLE-associated OD had a more severe and debilitating impact on all aspects of the patient’s quality of life than SLE prior to OD development, including further limitations in activities of daily living after the development of OD. **Discussion:** Study findings guided the development of a comprehensive conceptual model that fully represents the patient experience of living with SLE-associated OD, highlighting the additional burden of OD when compared with SLE alone. **Conclusions:** The conceptual model will inform improvements in disease management, which may result in better patient outcomes and aid development of clinical outcome assessments of disease burden.

## BACKGROUND

Systemic lupus erythematosus (SLE) is a chronic autoimmune inflammatory disease that affects many organ systems (eg, musculoskeletal, mucocutaneous, and renal) and is associated with reduced health-related quality of life (HRQoL) and increased morbidity and mortality.^1–3^ Patients with SLE, particularly those with uncontrolled disease activity or who have been treated for prolonged periods with high-dose corticosteroids, are at higher risk of developing irreversible organ damage (OD)^2,4–8^; within 10 years of an SLE diagnosis, half of patients develop OD.^9^ Once present, the risk of damage accrual is also influenced by several non-SLE associated factors, including health status, age, sex, race, ethnicity, and socioeconomic status.^6–8,10,11^

It has been well documented and defined by the Systemic Lupus International Collaborating Clinics/American College of Rheumatology (SLICC/ACR) Damage Index (SDI) that OD may involve the cardiovascular, dermatological, gastrointestinal, musculoskeletal, neuropsychiatric, ocular, peripheral vascular, pulmonary, and renal systems; additionally, patients experience OD-related diabetes, malignancy, and premature gonadal failure.^12^ As a result, there is a substantial humanistic and economic burden associated with SLE-associated OD. Patients with SLE-associated OD also experience diminished HRQoL outcomes, particularly in terms of physical functioning,^13,14^ and substantially increased healthcare expenditure.^15–19^ Ten-year cumulative healthcare costs for patients with SLE-associated OD (SDI score ≥5) are almost 9 times higher than those reported for patients with no OD (SDI = 0)^18^; with some forms of OD (such as cardiovascular, neuropsychiatric, and renal) having a greater impact on healthcare expenditure compared with damage in other organ systems.^15,16^

Previous publications have documented the patient burden of SLE^20,21^ and the importance of collecting patient-reported outcomes data in SLE clinical trials^22^; however, the overall impacts of SLE-associated OD have not yet been fully characterized. Given the growing importance of patient-focused drug development, gathering a more robust understanding of the patient experience of SLE-associated OD is necessary.^23^

This qualitative study aimed to characterize the patient experience of SLE-associated OD, including impacts on functional status, financial status, employment, and the overall burden and impacts of treatment. These findings were then used to develop a conceptual model representing the patient experience of living with SLE-associated OD.

## METHODS

### Study Design

This was a qualitative, noninterventional study with three distinct phases (GSK Study 209754): (1) a targeted literature review; (2) concept elicitation interviews with key opinion leaders (KOLs); and (3) concept elicitation interviews with patients diagnosed with SLE-associated OD.

Informed consent was obtained from all eligible participants included in the study. All study materials were reviewed and approved by the New England Independent Review Board (NEIRB; No. 120190518).

### Targeted Literature Review

A targeted literature search, limited to English-language articles, was conducted in PubMed and the Lupus Science and Medicine database between June 19 and July 3, 2019, to establish the humanistic and economic burden of SLE-associated OD, treatment outcomes, and use of conceptual models (**Supplementary Table S1**). All abstracts were screened and relevant articles selected for full-text review (**Supplementary Figure S1**). Additionally, reference lists of key articles and articles known to the authors were reviewed for relevance. The literature review findings informed the development of a draft conceptual model of the impact of SLE-associated OD and interview guides.

### Recruitment of KOLs

KOLs were either clinicians who were experienced in working with patients with SLE and SLE-associated OD or patient advocacy leaders. An email invitation was sent and interested KOLs were scheduled for a one-on-one telephone interview. A copy of the draft conceptual model was shared prior to the interview.

### Recruitment of Patients

Patients were recruited through collaboration with a third-party recruitment agency that specializes in identifying patients for noninterventional, qualitative research studies. Potential participants were identified from an existing list of individuals who had previously volunteered to take part in this type of research, recruiter databases, physician referrals, patient support groups, and social media (eg, Facebook) in the United States (US). Patients were eligible to take part if they were at least 18 years of age; fluent in spoken and written US English; lived in the US; had confirmed diagnoses of long-term OD associated with SLE; and had evidence of damage in at least 1 organ system (cardiovascular, dermatological, diabetes, gastrointestinal, malignancy, musculoskeletal, neuropsychiatric, ocular, peripheral vascular, premature gonadal failure, pulmonary, and renal) as defined by the SDI.^12^ Patients’ SLE diagnosis was confirmed by (1) physicians completing the confirmation form, (2) patients providing a screenshot or printout of their diagnosis from a patient portal or medical record, or (3) patients providing a photo of their current medications for the treatment of SLE.

Patients were excluded if they were unable to independently participate in a 60- to 75-minute telephone interview.

### Concept Elicitation Interview Procedures

Concept elicitation is a qualitative research method that allows the patient experience to be explored in depth, typically via one-on-one interviews or focus groups. This type of interview is recommended by the US Food and Drug Administration to “identify what is most important to patients with respect to their experience as it relates to burden of disease and burden of treatment.”^23^ Translating these qualitative data into a conceptual model allows the patients’ experiences to be distilled into a graphic display of how the patients described living with this condition.^24^

Phone and/or webcam interviews with KOLs and patients were conducted by experienced qualitative researchers using semi-structured interview guides designed to allow interviewees to share their opinions and experiences.

### KOL Interviews and Analysis

Interviews with KOLs lasted 60 minutes and covered:

- Their background and experience treating patients with SLE-associated OD, including their perspective on the burden experienced by patients
- Their feedback on the draft conceptual model, including the clarity, language, comprehensibility, and potential use of the conceptual model
- The extent to which the draft conceptual model reflects the patient experience and any concepts that are missing or should be removed; considerations for the patient interviews
- Any additional feedback

All interviews were audio recorded and transcribed verbatim; all transcripts were quality checked by the research team for accuracy.

All interview transcripts were analyzed using thematic analysis in accordance with grounded theory wherein concepts emerge from data rather than researchers imposing a priori theory.^25–27^ Inductive coding was used to identify concepts and themes; all coding and analysis was conducted using NVivo 12 qualitative analysis software (QSR International Pty Ltd, Burlington, Massachusetts, USA).

KOL transcripts were coded by one researcher and the findings reviewed by a second for accuracy and consensus. Emergent themes were discussed and reviewed by the wider research team to provide feedback on the conceptual model and insights for developing the patient interview materials.

### Patient Interviews and Analysis

Patient interviews lasted 60 to 75 minutes and explored their experiences of living with SLE-associated OD. Participants were asked questions regarding the physical, emotional, social, and economic impact of SLE-associated OD on their quality of life and their experiences of treatment of SLE. Open-ended questions were followed by probing questions to clarify and assist interpretation of emerging concepts.

Similarly to the KOL interviews, all interviews were audio recorded and transcribed verbatim; all transcripts were quality checked by the research team for accuracy and analyzed using thematic analysis as described above.

The first 8 patient transcripts were each coded by 2 researchers to determine and refine the initial coding structure and ensure coding consistency. A third researcher participated in the review of coding (for consensus on coding and any new codes added) after the first 4 interviews were coded, and again when the next 4 had been coded. The 3 researchers split coding of the remaining 32 interviews, meeting when there were questions or clarification required. As additional patient interviews were coded and new themes or concepts arose, the coding schema was updated and refined to create a list of common themes that were eventually incorporated into the conceptual model. This process continued until no new concepts were identified. Saturation of concepts was achieved early in the data collection process, within the first 16 interviews; however, interviews continued to gain further valuable information including ensuring representation of each area of SLE-associated OD and representation of patients with a single area of OD, 2 areas of OD, and multiple areas of OD (≥3).

### Availability of Data and Material

Anonymized individual participant data and study documents can be requested for further research from https://www.gsk-studyregister.com/en/

## RESULTS

### Literature Review and Draft Conceptual Model

A total of 71 articles were retrieved for full-text review, including 2 articles describing existing conceptual models for SLE.^20,21^ Based on the literature review findings, to present a complete picture of the SLE-associated OD, the initial version of the conceptual model was compiled to: (1) include the overall impact of SLE and disease and economic impacts of SLE-associated OD; (2) show the relationships between SLE/OD and different treatments; and (3) incorporate clinical outcomes and treatment targets. Treatments and treatment targets are integrated parts of the disease model that inform better treatment decisions. This initial conceptual model was then reviewed by the KOLs.

### KOL Interviews

Six KOLs (5 clinicians and 1 patient advocacy leader [diagnosed with SLE in 1990]) were interviewed; 3 were from Europe, 2 from North America, and 1 from South America. Among the clinicians, 3 were rheumatology specialists, 1 was a rheumatologist/nephrologist, and 1 specialized in internal medicine. Their clinical experience of treating patients with SLE ranged from 11 to 21 years or more.

The KOLs reported that the draft model broadly captured the patient experience of SLE-associated OD; however, they offered suggestions to clarify concepts captured by the model. First, they emphasized the need to highlight that the impacts of SLE are manageable and reversible, while impacts of OD are irreversible and permanent (“… the point of no return means that from this [point on] it’s irreversible and it causes more damage or it will affect more organ systems” [ID 03, rheumatologist]). Next, they indicated that the conceptual model should clearly demonstrate that patients with SLE-associated OD experience more complications and impacts of disease (eg, increased risk and impact of comorbidities, psychological/cognitive damage, infertility, permanent work disability), compared with patients without SLE-associated OD. Additional recommendations are included in the supplementary information (**Table S2**).

### Patient Interviews

Forty individuals with SLE-associated OD participated in the interviews, the majority of whom were female (n = 38; 95%); the mean age was 43.4 years (**Table 1**). Over half of patients were white (n = 22; 55%), and a fifth of patients were of Black African ancestry (n = 8; 20%). Overall, 13 (32.5%) patients had received their SLE diagnosis at least 10 years prior. Many participants reported being diagnosed with SLE-associated OD in more than 1 area (n = 31; 77.5%). The most prevalent areas of OD were dermatological (n = 24; 60%), renal (n = 22; 55%), musculoskeletal (n = 20; 50%), and pulmonary (n = 18; 45%). The 2 areas of organ damage that participants reported as most bothersome were pulmonary (n = 11; 61% of those who reported pulmonary damage) and renal (n = 10; 45% of those who reported renal damage).

**Table 1.** Patient Demographics and Clinical History

**Characteristic**	**Total (n = 40)**
Gender, n (%)	
Female	38 (95)
Mean age, y	43.4
Race, n (%)	
White	22 (55)
Black African ancestry	8 (20)
Latinx	5 (12.5)
Native American	1 (2.5)
Other	1 (2.5)
≥2 identified	3 (7.5)
US region, n (%)	
Southeast	15 (37.5)
Southwest	8 (20)
Midwest	8 (20)
Northeast	6 (15)
West	3 (7.5)
Time since SLE diagnosis, n (%)	
0-3 y	11 (27.5)
4-5 y	7 (17.5)

Abbreviations: OD, organ damage; SLE, systemic lupus erythematosus.

### Experience of SLE-Associated OD Compared With SLE Before Development of OD

Across the interviews, patients indicated that the impacts of SLE-associated OD are more impactful and debilitating than those of SLE without OD. Verbatim example quotes describing the participants’ experiences before and after development of OD are shown in **Table 2**. Most participants (n = 39; 97.5%) described areas of their lives that were more severely affected since their diagnosis of OD. This was expressed plainly by one participant:

“… it’s a constant battle um… and you’re always fighting, there’s… there’s never an end to the organ damage… with the organs unfortunately they just pile on more medications and it just makes you feel even worse” [ID 030, female, OD: cardiovascular, dermatological, gastrointestinal, musculoskeletal].

Another described the change in their symptoms as they developed OD:

“When my organs start um going, you know, active and showing more damage, the symptoms become a different type… the pain is definitely worse…” [ID 014, female, OD: cardiovascular, dermatological, malignancy, musculoskeletal, premature gonadal failure, pulmonary, renal].

**Table 2.** Patient Descriptions of the Impact of SLE-Associated OD

**Topic**	**Example Patient Quote**
Physical functioning: Severe fatigue	“Fatigue is, is terrible… There are days when you wake up, you’re okay, you’re, you know, you’re doing your thing and then, it just kind of hits you like a… ton of bricks. And, you’re like, that’s it. I’m done. I’m gonna go lay down, and nobody bother me. And that’s just, kind of, how it goes. And you never know when this ton of bricks is gonna hit you.” *— ID 018, female; OD: dermatological, gastrointestinal, musculoskeletal, peripheral vascular, pulmonary*
Cognitive functioning	“… my brain is always super foggy, like, I used to pride myself on being super witty and, you know, being able to have a conversation that would change, you know, topics all the time and I could, I could keep up. I was a great conversationalist. And now, I honestly, I feel like somebody stole half my IQ… I used to be a pharmacy technician… If I gave them the wrong medication, or the wrong dose. Or didn’t check to see if there’s interactions… it is a lot of responsibilities and I loved my job, but it was getting to be… difficult… instead of double-checking every prescription, I was having to, you know, check five, six, seven times. And, I was having to have… my employees, the people that worked for me and under me, check my work for me”. *— ID 015, female; OD: cardiovascular, dermatological, musculoskeletal, ocular, renal*
Psychosocial functioning: Emotional	“I find myself getting more irritable, I find myself just waking up angry at life, some days I can be the strongest woman in the world and some days I want to stay a fetal position and cry, but I call those my soulless moments and that’s how I defeat that feeling so what? You have lupus, so what, you have organ damage, so what you’re going through these things? Look at the positive things you’ve achieved, you have five degrees, you have beautiful children, you have um… businesses that you have created on your own, you are fierce, you can do it, so I kind of encourage myself. Because at this point in my life, I feel confined, restricted, I feel less than a mother um, a spouse, I feel as if my life is deteriorating before my eyes and nobody can help me” *— ID 010, female; OD: cardiovascular, dermatological, gastrointestinal, musculoskeletal, neuropsychiatric, ocular, peripheral vascular, pulmonary, renal*
Psychosocial functioning: Social	“… it’s a fatigue in my body to the point where I feel like I can’t do something… it’s past exhaustion… I have never had this before I was diagnosed… I was tired, but I could function. This is sometimes to the point where I don’t feel I can function… I was uh real active in making crafts and doing things you know around my house um, gardening, stuff like that, the fatigue is so bad I don’t feel like I can do that anymore… as far as going out, I used to exercise a lot and I find that I’m… I just feel so tired that when my friends call me and say, ‘hey do you want to go walking?’ I can’t motivate myself to go walking because I just have… it’s like a heaviness and I can’t… I just can’t do it. And I’m afraid to commit to things because I’m afraid I’ll get like halfway through and then I’ll stop” *— ID 036, female, OD: dermatological, musculoskeletal, ocular, renal*
Psychosocial functioning: Relationships	“[My previous partner] was trying to push me more saying oh come on, you can go, go, go and then when he… saw that I physically had to sit down and I couldn’t do things, it changed the dynamics of our relationship… it’s changed our roles because he feels like he has to be a caregiver instead of my equal… and with my son um you know, we used to go to movies all the time um I, at one time was in law enforcement and he is now in law enforcement and we used to go to the range, you know and, and keep up our, our skills and I can’t do that anymore… So, it’s changed that dynamic as well” *— ID 009, female; OD: musculoskeletal, renal*
Economic and work- related well-being	“I did manage to keep working, you know, up until about 2010 and I just couldn’t do it anymore. I just couldn’t… And, you know, fortunately I managed to get my disability, a lot of people aren’t able to do it and you know, it’s… it’s difficult but um, I got it and then that was a huge lifestyle change again for me… I’m in uh public housing so… there’s no way that I could be able to live on my own, you know, out in the real world because it’s too expensive… I don’t know what I would do. You know, I get a whole whopping $16 in food stamps… that at times can be an issue because you know, you only have so much money to spend and then it’s gone so…um…you know, it can be an issue sometimes” *— ID 033, female; OD: gastrointestinal, peripheral vascular, pulmonary*
Overall impact of SLE- associated OD compared with SLE without OD	“It wasn’t that much of an issue before the organs were involved, um, but I say that looking back, I mean at the time I felt terrible, but now that I… once I developed the organ damage I look back, I’m like oh I wish I could go back to then” — *ID 041, female; OD: dermatological, musculoskeletal*

Abbreviations: OD, organ damage; SLE, systemic lupus erythematosus.

**Figure 1.**
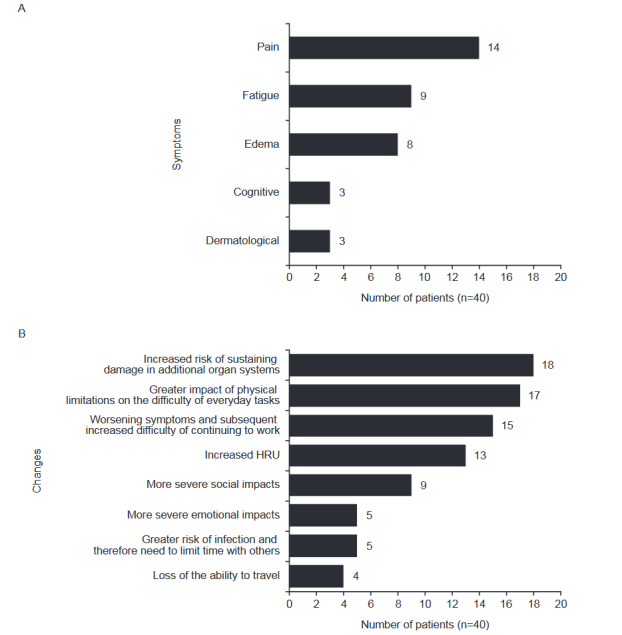
Symptoms Reported by Patients With SLE as More Severe (**A**) and Impacts Experienced (**B**) Following OD Diagnosis Abbreviations: HRU, healthcare resource utilization; OD, organ damage.

The increase in symptom severity with SLE-associated OD was a common theme across the interviews. Many participants noted that their clinical manifestations (especially pain, fatigue, edema, cognitive symptoms, and dermatological involvement) were more severe since being diagnosed with OD (n = 30; 75%) (**Figure 1A**). Participants described how the physical limitations of worsening symptoms made simple, everyday tasks (eg, dressing, cooking, and cleaning) more difficult. Relationships, careers, and mental health also deteriorated for many participants since being diagnosed with OD (**Figure 1B**). Participants reported how they had left their jobs since being diagnosed with OD, due to the difficulty of continuing to work with worsening symptoms or being advised that the stress of continuing to work would lead to more flares and increased risk of further OD. Others shared how they found it more difficult to attend social events and gatherings. For example, one participant avoided going out due to worsening symptoms and their distress about the changes in their physical appearance:

“… I don’t even want to, to go, if I’m having really bad hand sores, I don’t want to go out. I don’t want people to see my hands” [ID 021, female, OD: dermatological, musculoskeletal].

These feelings of distress, depression, and anxiety were common to all participants when comparing their lives before and after developing OD and, as one stated, “… it just changes everything in your life” [ID 030, female, OD: cardiovascular, dermatological, gastrointestinal, musculoskeletal].

### Impacts of SLE-Associated OD

Participants reported many impacts of SLE-associated OD, which were organized into four domains: physical functioning, cognitive functioning, psychosocial functioning, and economic and work-related well-being. Verbatim quotes describing the impacts of SLE-associated OD are shown in **Table 2**.

**Figure 2.**
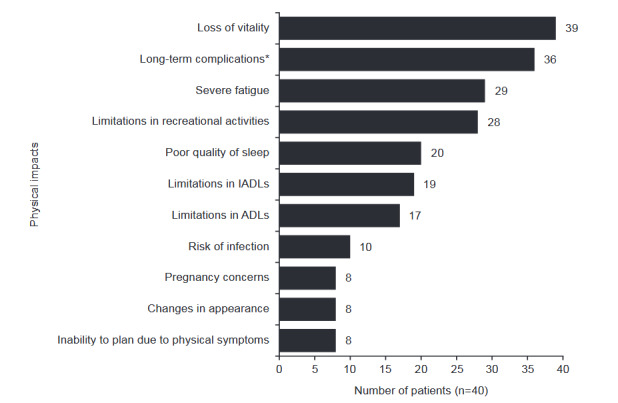
Impacts on Physical Functioning Caused by SLE-Associated OD as Reported by Patients *Includes gallbladder issues, diabetes, unstable blood pressure, extreme pain, poor mobility, edema, pleurisy, asthma, inflammation, dialysis, dietary issues, infections, and Raynaud’s disease or phenomenon. Abbreviations: ADLs, activities of daily living; IADLs, instrumental activities of daily living; OD, organ damage; SLE, systemic lupus erythematosus

**Impacts on physical functioning**: All participants described extreme daily physical impairment from SLE-associated OD and found these impairments to be especially debilitating. Patients across the interviews described 11 key impacts related to physical impairment, with loss of vitality, long-term complications (including gallbladder issues, unstable blood pressure, extreme pain, poor mobility, edema), severe fatigue, and limitations of recreational activities being the most common (**Figure 2**). Nearly all participants (n = 39; 97.5%) described an overall loss of vitality. They experienced an inability to participate in life as they had before OD and an overall lack of energy or liveliness, which overlapped with the more specific physical impacts of fatigue and long-term complications. These impacted their ability to perform simple, everyday tasks with one participant explaining:

“I can’t clean for long periods of time, I can’t bathe, you know, get in and out the tub without being out of breath, I can’t get out the bed without feeling like my body wants to shut down sometimes…” [ID 010, female, OD: cardiovascular, dermatological, gastrointestinal, musculoskeletal, neuropsychiatric, ocular, peripheral vascular, pulmonary, renal].

**Impacts on cognitive functioning:** Almost three-fourths of patients (n = 27; 67.5%) readily described experiencing changes in their cognitive functioning, commonly referring to it as “brain fog.” Participants described these changes as slower cognitive processing and/or decision-making skills, forgetfulness or memory problems, confusion, and aphasia/difficulty with word recall. One participant shared:

“… I found myself mentally slowing down. It took me a little longer to uh, to comprehend things, sometimes I’d have to read it over, three or four times, to be able to comprehend…” [ID 037, female, OD: dermatological, pulmonary].

Often, these impacts overlapped with emotional impacts (increased frustration or anger) and work impacts (reduced ability to do their job).

**Impacts on psychosocial functioning:** Almost all participants (n = 39; 97.5%) shared how living with SLE-associated OD had affected them emotionally, reporting anxiety, depression, frustration, fear, stress, anger, helplessness, and mood changes. One participant reported:

“… I could be fine one minute of the day and then all of a sudden … the depressed, hopeless, helpless feeling becomes overwhelming…” [ID 014, female, OD: cardiovascular, dermatological, malignancy, musculoskeletal, premature gonadal failure, pulmonary, renal].

Participants were generally worried about their future and the unpredictability of OD, and often felt embarrassed and stigmatized because of their condition. Emotional impacts were also evident when discussing social lives and relationships, all participants (n = 40; 100%) reported that SLE-associated OD had significantly affected their ability to socialize; one participant stated:

“… it’s really impacted me because I’m basically a hermit. I don’t leave my house” [ID 024, female, OD: dermatological, musculoskeletal, diabetes, premature gonadal failure, pulmonary, renal].

Participants described how stepping back from meaningful social connections had led to feelings of isolation and how their need to do so was linked to physical symptoms of OD, risk of infections, sensitivity to sun/extreme temperatures or an inability to make plans because of unpredictable symptoms. SLE-associated OD complicated participants’ family relationships (n = 30; 75%). They spoke of the difficulties they and their families encountered when their physical health deteriorated and their need to rely on others increased, their feelings of being a burden to their families, and changes in their relationship dynamics. As participants may outwardly appear healthy, many of those interviewed felt misunderstood by family and friends who lacked understanding of what it means to live with OD and the debilitating nature of symptoms, further complicating relationships. Issues of intimacy, including physical intimacy, within relationships were also highlighted by interviewees with partners whilst others were reluctant to pursue a new relationship because of the complications associated with OD.

**Impacts on economic and work-related well-being:** All participants experienced or expressed concern about the economic impacts of SLE-associated OD. Economic impacts included inability to work, underemployment, costs of care and medications, loss of health and/or life insurance, and increased nonmedical costs. Specific work-related impacts (lost productivity, increased absenteeism, or lost career path/aspirations) were experienced or were a concern for most participants (n = 38; 95%) regardless of current employment status. Notably, many participants (n = 31; 77.5%) shared that they were no longer working because of their SLE-associated OD. For those still working, the symptoms they experience played a role in lost productivity and absenteeism, distracting them or necessitating time off if they were not feeling well. Participants also reported they abandoned career aspirations or altered career plans following their diagnosis of OD, and many felt apprehensive about seeking new or different jobs because of the workplace accommodations that would be needed.

### Treatment Goals

Participants’ treatment goals closely aligned with the ways in which SLE-associated OD has impacted their lives. Treatment goals were distilled into 3 categories: disease management (n = 25; 62.5%; managing symptoms, minimizing and preventing flares, and avoiding long-term invasive treatments, eg, dialysis), improved HRQoL (n = 11; 27.5%; improving overall daily functioning, reducing the severity of impacts of SLE-associated OD, and returning to work), and medication use (n = 7; 17.5%; stopping corticosteroids, stopping or reducing all medications, and reducing the negative impacts of treatment).

### Final Conceptual Model

**Figure 3.**
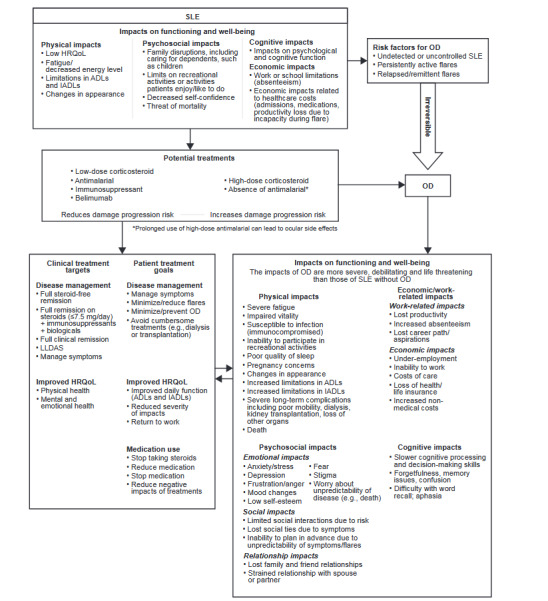
Final Conceptual Model Abbreviations: ADLs, activities of daily living; HRQoL, health-related quality of life; IADLs, instrumental activities of daily living; LLDAS, lupus low disease activity state; OD, organ damage; SLE, systemic lupus erythematosus.

Following patient interviews, the draft conceptual model was modified to reflect the patient experience of SLE-associated OD more accurately. The impacts of SLE and SLE-associated OD (physical, cognitive, psychosocial, and economic and work-related) were reorganized to fall under functioning and well-being domains. Further revisions to the draft model included: clarification of risk factors for SLE-associated OD; placing further emphasis on the permanent and irreversible nature of OD; redefinition and streamlining of clinical treatment targets and patient treatment goals; and representation of treatments on a simple spectrum from slowing damage progression to increasing risk of damage progression. The final conceptual model is displayed in **Figure 3**.

## DISCUSSION

In this study, findings from the targeted literature review and concept elicitation interviews with 6 KOLs and 40 patients in the US guided the development of a comprehensive conceptual model that fully represents the patient experience of living with SLE-associated OD. Consideration of data from a sample of patients with damage across various organ systems, different levels of damage, and significant variation in the time since diagnosis ensured the model reflected the experiences of a broad range of patients.

The conceptual model displays the impacts of SLE-associated OD on 4 distinct areas of patients’ lives: physical, cognitive, and psychosocial functioning, and economic and work-related well-being. Other studies have also shown that increased OD negatively impacts patients’ emotional, physical, and social functions, as measured by the Medical Outcomes Study Short Form-36.^8,14^ Physical impacts were especially devastating to patients and overlapped with impacts in all other areas of patients’ lives. For example, patients shared how physical symptoms impacted their ability to work, while others commented that their social lives and relationships had been negatively affected by the physical aspects of their condition. Nearly all patients reported a loss of vitality that overlapped with more specific impacts on their everyday life, such as fatigue. About three-fourths of patients described problems with cognitive functioning, which were closely tied to emotional and work-related impacts. All patients reported concerns about the economic consequences of SLE-associated OD, including impacts on employment and ability to work, and increased medical costs.

A key finding of this study was that the impacts and patient burden of OD are far greater than those associated with SLE prior to OD development. Many patients experienced more severe symptoms and impacts including physical impairment, significant changes in their relationships, employment status, and feelings of anxiety and depression. As one patient commented, “… it just changes everything in your life…” This notable increase in burden, which is also supported by previous studies,^13,14,28^ highlights the permanent and irreversible nature of SLE-associated OD and emphasizes the importance of slowing OD progression. This is consistent with the recent inclusion of prevention of OD as a treatment goal in clinical guidelines and further emphasizes the value of this treatment goal for patients living with SLE-associated OD.^29^ Key treatment goals identified by the patients in this study were to reduce disease activity, manage symptoms, reduce medication use, and improve HRQoL.

Understanding the patient experience of a disease is essential for improving clinical practice and research. By focusing on the patient experience of SLE-associated OD, this study built upon existing conceptual models depicting the burden of SLE where OD was not the focus^20,21^ and expanded our understanding of how different the experience and impacts of OD are (including risk factors for OD, treatment management, and experiences). Although the themes may not be unique to OD compared with patients with SLE, KOLs highlighted that patients with SLE-associated OD had a higher rate of mortality, and patients reported experiencing further limitations in ADLs and lower HRQoL after the development of OD.

A limitation of this study was the lack of a control group with SLE and no OD. Participants retrospectively compared their experiences before and after the onset of SLE-associated OD and reported more severe impacts across more areas of functioning and well-being since incurring OD. However, given the impacts on functioning and well-being are similar to those reported by patients with SLE and no OD, a control group would allow for further distinction in the severity of impacts. In addition, SLE is known to disproportionately affect non-white individuals who experience a more rapid accumulation of damage,^30,31^ so the extent to which these findings can be generalized to all SLE patients might be limited as more than half of patients were white. Furthermore, most patients were from the Southeast region of the US, and the results may reflect more of their experience than the experience of those from other regions of the US. In future studies, it would also be interesting to compare the experiences of patients with SLE and newly diagnosed OD and those with longer-term OD (ie, >10 years), and to understand the experiences of patients with SLE and OD with and without health insurance.

## CONCLUSIONS

This comprehensive conceptual model represents the patient experience of living with SLE-associated OD, highlighting the additional burden of OD when compared with SLE alone. The model will aid in the development of clinical outcome assessments (eg, patient-reported outcome instruments) of disease burden and will inform future improvement in disease management and therefore outcomes of these patients.

### Author Contributions

All authors contributed to the conception and design of the study and data analysis or interpretation. L.B., A.F., and C.U.F. also contributed to data acquisition. All authors have made substantial contributions to the development of the manuscript and approved the final version.

### Disclosures

L.B. and A.M.F. are current employees of QualityMetric, and C.U.F. is a former employee of QualityMetric. QualityMetric received funding from GSK to conduct this research. W.H.C., R.A.L., and D.C. are employees of GSK and hold stocks and shares in the company. K.G. is a former employee of GSK and holds stocks and shares in the company.

### Presentation

Results of this study were presented at the EULAR 2021 Congress.

## Supplementary Material

Supplementary Online Material
