# Supplementary material for: Development of a Conceptual Model to Understand Disease Burden in Patients With Systemic Lupus Erythematosus and Organ Damage
Source: J Health Econ Outcomes Res. 2023 Aug 18;10(2):30–8. doi: 10.36469/001c.82228 (PMC10440069; doi:10.36469/001c.82228)
Supplement: Supplementary Online Material [file jheor_2023_10_2_82228_178476.pdf]

Journal of Health Economics and Outcomes Research

Online Supplementary Material

Development of a Conceptual Model to Understand Disease Burden in Patients With Systemic Lupus Erythematosus and Organ Damage. *JHEOR*. 2023;10(2):30-38. doi:10.36469/jheor.2023.82228

Table S1: Search Strings for Systematic Searches Conducted in PubMed and Lupus Science and Medicine Database

Figure S1: Flow Diagram of the Literature Review Process

Table S2: Additional Key Opinion Leader Recommendations for Revisions to the Draft Conceptual Model

This supplementary material has been provided by the authors to give readers additional information about their work.

This is an open-access article distributed under the terms of the Creative Commons Attribution 4.0 International License (CCBY-4.0). View this license's legal deed at http://creativecommons.org/licenses/by/4.0 and legal code at http://creativecommons.org/licenses/by/4.0/legalcode for more information.

Table S1. Search Strings for Systematic Searches Conducted in PubMed and Lupus Science and Medicine Database

PubMed Search Strings

Disease String	Model/Impact Strings
(“SLE” OR “lupus” OR “systemic lupus erythematosus”) AND (“long term organ damage” OR “damage accrual” OR “LTOD”) ***AND***	1. “conceptual model”
	2. “burden” OR “disease burden” OR “burden of disease” OR “patient burden” OR “patient impact” OR “burden of illness” OR “quality of life” OR “impact”
	3. “economic burden” OR “economic impact” OR “cost” OR “cost of illness” OR “productivity” OR “work” OR “employment” OR “healthcare cost” OR “healthcare utilization” OR “absenteeism” OR “presenteeism” OR “economic” OR “financial” OR “medical cost” OR “hospitalization”
	4. “treatment benefit” OR “outcomes of treatment” OR “prevention” OR “reduc*” OR “benefit” OR “remission” OR “low disease activity”
	5. “quality of life” OR “QOL” OR “HRQoL”

Lupus Science and Medicine Strings

“quality of life” OR “QOL” OR “HRQoL” AND “long term organ damage” OR “damage accrual” OR “LTOD”

Abbreviations: HRQoL, health-related quality of life; LTOD, long-term organ damage; QoL, quality of life; SLE, systemic lupus erythematosus.

Figure S1. Flow Diagram of the Literature Review Process

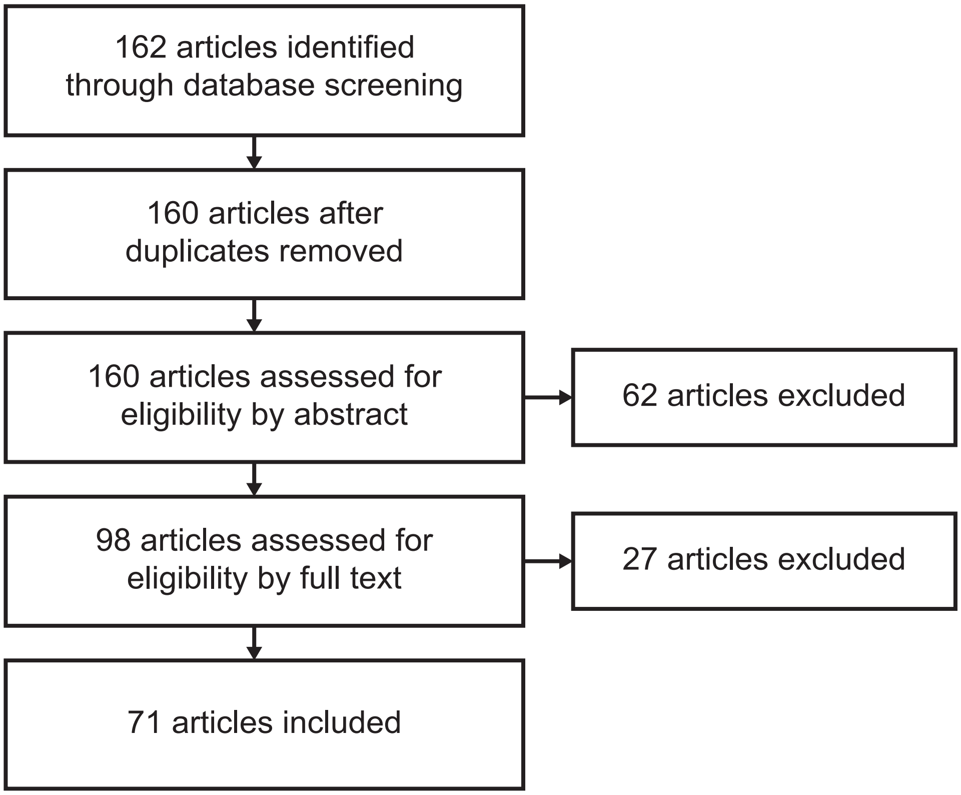

Table S2. Additional Key Opinion Leader Recommendations for Revisions to the Draft Conceptual Model

**Recommendations**
• Improve clarity by specifying the impacts of different areas of OD<br>• Provide further clarification or an improved definition of concepts such as HRQoL (eg, defining low HRQoL as impacts on energy levels and daily activities, family life and relationships, family planning, recreational activities, and appearance/self-esteem) and economic impacts<br>• Clarify the increased risk of mortality associated with OD<br>• Reorganize the presentation of the different treatments to acknowledge that although steroids become dangerous at high doses for long periods of time, they are quite helpful at lower doses, and that antimalarials may lead to OD under certain circumstances<br>• Adjust some of the clinical outcomes and treatment goals, including the addition of improvement of HRQoL as a treatment goal

Abbreviations: HRQoL, health-related quality of life; organ damage.
